# Bioelectromagnetics Research within an Australian Context: The Australian Centre for Electromagnetic Bioeffects Research (ACEBR)

**DOI:** 10.3390/ijerph13100967

**Published:** 2016-09-29

**Authors:** Sarah P. Loughran, Md Shahriar Al Hossain, Alan Bentvelzen, Mark Elwood, John Finnie, Joseph Horvat, Steve Iskra, Elena P. Ivanova, Jim Manavis, Chathuranga Keerawella Mudiyanselage, Alireza Lajevardipour, Boris Martinac, Robert McIntosh, Raymond McKenzie, Mislav Mustapic, Yoshitaka Nakayama, Elena Pirogova, M. Harunur Rashid, Nigel A. Taylor, Nevena Todorova, Peter M. Wiedemann, Robert Vink, Andrew Wood, Irene Yarovsky, Rodney J. Croft

**Affiliations:** 1 Australian Centre for Electromagnetic Bioeffects Research, Wollongong 2522, Australia; s3507715@student.rmit.edu.au (A.B.); mark.elwood@auckland.ac.nz (M.E.); John.Finnie@sa.gov.au (J.F.); Steve.Iskra@team.telstra.com (S.I.); eivanova@swin.edu.au (E.P.I.); ckeerawella@swin.edu.au (C.K.M.); alajevardipour@swin.edu.au (A.L.); B.Martinac@victorchang.edu.au (B.M.); Robert.L.McIntosh@team.telstra.com (R.McI.); Ray.McKenzie@amta.org.au (R.McK.); elena.pirogova@rmit.edu.au (E.P.); nigel_taylor@uow.edu.au (N.A.T.); nevena.todorova@rmit.edu.au (N.T.); peter_wiedemann@uow.edu.au (P.M.W.); robert.vink@adelaide.edu.au (R.V.); awood@swin.edu.au (A.W.); irene.yarovsky@rmit.edu.au (I.Y.); rcroft@uow.edu.au (R.J.C.); 2 School of Psychology and Illawarra Health & Medical Research Institute, University of Wollongong, Wollongong 2522, Australia; 3 Institute for Superconducting and Electronic Material (ISEM), University of Wollongong, Wollongong 2522, Australia; shahriar_hossain@uow.edu.au (M.S.A.H.); josip_horvat@uow.edu.au (J.H.); mislav@uow.edu.au (M.M.); 4 School of Engineering, RMIT University, Melbourne 3001, Australia; harun.rashid@rmit.edu.au; 5 School of Population Health, University of Auckland, Auckland 1072, New Zealand; 6 SA Pathology, Hanson Institute, Centre for Neurological Diseases, and School of Medicine, University of Adelaide, Adelaide 5000, Australia; jim.manavis@adelaide.edu.au; 7 Chief Technology Office, Telstra Corporation, Melbourne 3000, Australia; 8 School of Health Sciences, Swinburne University of Technology, Melbourne 3122, Australia; 9 School of Science, Swinburne University of Technology, Melbourne 3122, Australia; 10 Victor Chang Cardiac Research Institute, Darlinghurst 2010, Australia; Y.Nakayama@victorchang.edu.au; 11 Australian Mobile Telecommunications Association, Canberra 2603, Australia; 12 Centre for Human and Applied Physiology, School of Medicine, University of Wollongong, Wollongong 2522, Australia

**Keywords:** bioelectromagnetics, EMF, RF bioeffects, research

## Abstract

Mobile phone subscriptions continue to increase across the world, with the electromagnetic fields (EMF) emitted by these devices, as well as by related technologies such as Wi-Fi and smart meters, now ubiquitous. This increase in use and consequent exposure to mobile communication (MC)-related EMF has led to concern about possible health effects that could arise from this exposure. Although much research has been conducted since the introduction of these technologies, uncertainty about the impact on health remains. The Australian Centre for Electromagnetic Bioeffects Research (ACEBR) is a National Health and Medical Research Council Centre of Research Excellence that is undertaking research addressing the most important aspects of the MC-EMF health debate, with a strong focus on mechanisms, neurodegenerative diseases, cancer, and exposure dosimetry. This research takes as its starting point the current scientific status quo, but also addresses the adequacy of the evidence for the status quo. Risk communication research complements the above, and aims to ensure that whatever is found, it is communicated effectively and appropriately. This paper provides a summary of this ACEBR research (both completed and ongoing), and discusses the rationale for conducting it in light of the prevailing science.

## 1. Introduction

With over 6 billion mobile phone subscriptions worldwide, and the rise in use of other technologies such as Wi-Fi and smart meters, the electromagnetic fields (EMF) that power these technologies is now ubiquitous. This dramatic increase in use and community exposure to EMF has led to concern about possible health effects, as well as increased demand for scientific research. This desire for evidence-based information relates to not only the primary radiofrequency (RF; 3 kHz–300 GHz) fields emanating from these devices, but also the RF pulse-sequence or modulation frequency (which can be <3 kHz) and other related fields such as those due to battery currents, and whether they may independently or conjointly affect health. We shall refer to this broad nexus of mobile communications (MC)-related EMF as MC-EMF.

There has been a substantial body of research conducted to address the potential for adverse health and biological impacts of exposure to MC-EMF, with the predominant view being that MC-EMF bioeffects are primarily thermal, and that unlike the damaging effects of “high-level” RF (such as those that occur within microwave ovens), there is no substantiated evidence that these bioeffects adversely affect health at the relatively low levels (hereafter “low level”) associated with MC [1]. However, given the large proportion of the population exposed to MC-EMF, as well as strong community concern, further research would appear an appropriate prudent measure at this juncture. Accordingly, the World Health Organization (WHO) has published their 2010 RF Research Agenda, which tries to promote research in a number of areas that have relevance to public health in order to reduce scientific uncertainties and respond to public concern [2]. Relevant to the present review, this agenda includes, (1) epidemiological studies to identify potential behavioural and neurological disorders associated with exposure, as well as cancer and brain tumour incidence trends; (2) human studies focusing on the mechanisms, thresholds, and dose–response relationships of exposure-induced changes on electrical brain activity (electroencephalogram, EEG); (3) animal studies on development, behaviour, neurodegenerative disease, and reproduction; (4) cellular studies; (5) dosimetry studies, particularly in relation to quantifying personal exposures to MC-EMF; and (6) social science studies, dealing with how potential risks are communicated, as well as whether people’s perception of MC-EMF health risk can affect their wellbeing (e.g., electromagnetic hypersensitivity).

The Australian Centre for Electromagnetic Bioeffects Research (ACEBR) is a National Health and Medical Research Council (Australia) Centre of Research Excellence (2013–present), with its main aim to address the WHO’s research recommendations in order to better understand potential health effects of MC-EMF exposure. This involves research domains ranging from epidemiology, in vivo experiments (human and nonhuman), in vitro, and all-atom modelling, to the requisite dosimetry that underpins all Centre projects. As such, it represents an opportunity to review the current state of a variety of mobile communication EMF issues, outlining the current state of science, the justification for the related Centre research and its relevance to the MC-EMF health debate. Where data from Centre research have been obtained, either preliminary or completed peer-reviewed publications, this will also be provided. Note that unpublished data is described to provide an indication of the direction that the research is taking; it must be considered tentative, and as with all reports, considered in light of the extant literature.

It should be noted that in apparent contradiction to the prevailing scientific position described above, there are a large number of reports of alternative EMF–bioeffect interaction mechanisms and health effects themselves, and accordingly, a number of scientists who reject the standard science position. For example, a petition has recently been submitted to the United Nations, signed by over 200 scientists, requesting an urgent reassessment of the prevailing science and associated public health standards (www.emfscientist.org). This review will not address that debate, but rather bases its discussion of the research from the point of view of the prevailing scientific position.

## 2. Brain Tumour Incidence

One of the largest concerns in recent times has been whether mobile phone use increases the risk of brain cancer. A number of studies have addressed this, including two major cohort studies, which showed no increased risk [3,4], and several case-control studies, which have generally provided inconsistent results [5]. The largest, the Interphone study, showed no total increase in risk and no dose–response, but showed a 40% increase in glioma in the highest category of cumulative call time, an effect which the study authors concluded was likely due to bias [6]. However, although most studies have shown no clear increased risk, a set of case-control studies from Sweden (e.g., [7]) has reported increased risks, including risks within a few years from first phone use.

Methodological limitations with case-control studies, such as the potential for recall bias, can limit the interpretability of results and may explain the conflicting reports. Studying time trends in glioma incidence offers a complementary approach that can determine whether case-control reports of increased risk are consistent with the patterns seen in the general population; if phone use conferred a substantially increased risk within 10–15 years of first regular use, an increase in the population incidence of glioma (a tumour arising within the glial cells) would by now be apparent. To test this, we assessed glioma incidence trends from 1995 to 2010 in New Zealand, by age group and gender, and also by site of origin, as RF from mobile phones mainly reaches the parietal and temporal lobes [8]. No consistent increases in tumour incidence were observed; indeed, the incidence in the 10–69 years age group reduced over time, providing strong evidence against there being a substantial increase within a few years of starting phone use. Despite this, it should be noted that an effect with a latency period of more than 10–15 years, a very small effect, or one restricted to a rare subtype of brain tumour, cannot be excluded. Incidence rates did increase in those over age 70, but this increase started before mobile phones were introduced, and is likely due to diagnostic improvements. A recent analysis of total brain cancer incidence in Australia also showed no consistent increases from 1982 to 2012 in any age group under age 70 [9]. Taken together, these studies suggest that there is no increased risk of brain tumours at least up to 15 years after first use. The Centre is now extending its time-trend research to assess incidence rates in Australia within subcategories of brain tumour.

## 3. Determinants of RF Health Concern (IEI-EMF)

There is a significant proportion of the population that suffers severe physical and psychological illness that they attribute to MC-EMF exposure, a condition known as idiopathic environmental intolerance attributed to electromagnetic fields (IEI-EMF). It is characterised by a variety of nonspecific symptoms (e.g., headaches, nausea, tingling, difficulty sleeping) when in proximity to devices that emit MC-EMF. There is currently no substantiated evidence that the symptoms are related to the EMF exposure [10], suggesting that it may be caused by perceptions or beliefs about the exposure (either conscious or subconscious): the so-called nocebo effect. Whether EMF plays a causal role in IEI-EMF is crucial in terms of treatment options, and the Centre has thus focused on determining whether, as is often claimed by IEI-EMF sufferers, the lack of evidence for a causal role is due to methodological difficulties.

The ACEBR is addressing this issue by testing self-reported IEI-EMF individuals using a double-blind provocation design, while accounting for purported methodological limitations. It (1) uses an idiographic approach to account for EMF-induced responses in particular individuals potentially being “washed out” in group analyses; (2) tests in an environment where the participant feels safe and asymptomatic (such as their own home) to minimise confounds such as stress and unplanned encounters with MC devices en route to the testing facility; (3) evaluates individually determined symptoms to ensure relevance to the individual; (4) uses an initial open-label trial to ensure that the EMF signal used (or belief about the signal used) is sufficient to produce the reported symptoms; and (5) uses sufficient replications (6 sham, 6 exposure) to enable statistical analysis within individuals. This research has proven difficult as it requires individuals to subject themselves to what they perceive as the cause of serious impairment, and to do so repeatedly over a 3 day period. Three IEI-EMF individuals have been assessed to date, with more planned if recruitment is possible. We have not found evidence that MC-EMF caused symptoms in any of the individuals, whereas belief was found to be a strong predictor of symptoms.

## 4. Mechanisms of Interaction

EMF can affect the body via electrostimulation (up to about 20 MHz), electroporation (when EMF energy is focused as contact currents), and heating (EMF causes molecules to vibrate more rapidly, which causes friction and generates heat). However, heating is the only known interaction mechanism within the frequencies and magnitudes relevant to mobile telecommunications. Due to this, current international exposure guidelines (such as those set by the International Commission on Non-Ionizing Radiation Protection, ICNIRP [11]) set restrictions based primarily on thermal interactions and effects. However, there is now substantiated evidence that low-level RF-EMF has an impact on the electroencephalogram (EEG, a measure of brain electrical activity), with the mechanism for this effect, as well as any potential functional consequences of it, unknown. As there is currently no strong evidence of nonthermal mechanisms, it is assumed that a thermal mechanism must underlie these effects. However, the possibility of a nonthermal mechanism cannot be discarded, and brings into question the claimed assurance of protection provided by current guidelines. A major focus of ACEBR research is thus whether low-level effects can be explained by thermal mechanisms, and whether there are additional nonthermal interaction mechanisms.

### 4.1. Human Studies

Studies have consistently shown that RF-EMF, such as that emitted by mobile phone handsets, has an impact on the EEG. This occurs during both awake [12,13] and sleep states [14,15], has been replicated numerous times [16,17,18], is consistent within individuals [16], and is potentially dose-dependent [19]. However, although biophysics suggests that this interaction with the brain must be thermal, the maximum cortical temperature elevation due to a mobile phone is only approximately 0.1 °C, which appears to be far too small to affect thermoregulation or the EEG. This dilemma is difficult to resolve, given that the thermal threshold for either an EEG change or a biologically relevant increase in thermoreceptor firing rate is not known.

The Centre is thus conducting research to determine whether the MC-EMF-induced EEG changes are thermally mediated. This involves exposing human participants to an MC-EMF signal emitted by a planar patch antenna, while clamping whole-body temperature (a combination of deep-body and skin temperatures) in a thermally neutral state using a water-perfused suit. This opens the thermoregulatory control loops, which is essential to prevent variations in thermoreceptor feedback and the corresponding modulation of sympathetic flow to the cutaneous vasculature and sweat glands [20]. This feedback has a powerful influence over cutaneous blood flow in the hands and the feet under thermoneutral conditions, and which, without clamping, can confound results.

In support of the mechanism being thermal, our first study found that local MC-EMF exposure within ICNIRP [11] general public restrictions is sufficient to generate a thermoregulatory response (increased peripheral blood flow to the finger), which means that MC-EMF could potentially affect the EEG via thermoregulatory change. A suite of studies is now testing whether (non-EMF) thermal profiles in the body, equivalent to those generated by RF-EMF, cause similar EEG changes.

### 4.2. In Vitro Receptor Sensitivity

Biomagnetic effects are not known to play major physiological roles in living organisms. However, given the above-described EMF-induced EEG changes, and claims of “nonthermal” impacts of RF-EMF on health more generally (e.g., [16,19]), it is important to revisit this issue and increase our understanding of magnetic field influences at the membrane, cell, and tissue levels. Further, studying static fields can be particularly useful for understanding potential nonthermal effects, as they do not result in significant heat production.

Membranes of human cells (and of most living organisms) are largely made of phospholipids as constituents of the membrane lipid bilayer. Phospholipid molecules forming the lipid bilayer can align themselves perpendicular to strong homogeneous magnetic fields (~1 T) because they possess diamagnetic anisotropy (a difference between parallel and perpendicular magnetic susceptibility). However, the physical structure of the lipid bilayer of cellular membranes has the potential to be influenced by moderate (~100 mT) intensity static magnetic fields (SMFs). These moderate SMFs can result in a rotational displacement of the membrane’s phospholipid molecules by virtue of their collective diamagnetic properties, which influence embedded membrane proteins, including ion channels. Thus, whereas it is not possible to orient individual phospholipid molecules in a magnetic field because of thermal fluctuations, phospholipids in ordered structures such as the lipid bilayer become oriented in moderate magnetic fields due to the summation of the diamagnetic anisotropy over a very large number of oriented molecules. Consequently, as a major component of biological membranes, the lipid bilayer presents a possible target of influence for magnetic fields.

ACEBR has been addressing this issue using mechanosensitive (MS) channels, membrane proteins that respond to mechanical stimuli that cause deformation of cellular membranes. Since MS channel function is closely related to the physical properties of the membrane lipid bilayer [21], these channels present good candidate molecules for detection of mechanical forces exerted onto biological membranes under the ordering influence of magnetic fields [22]. Given that their malfunction underlies pathology of diseases such as cardiac hypertrophy and arrhythmias, neuronal degeneration, muscular dystrophy, xerocytosis (familial anemia), and arthrogryposis (congenital joint contractures), the moderate static as well as electromagnetic fields could potentially exert adverse effects on living cells by affecting the function of MS channels.

**Figure 1:**
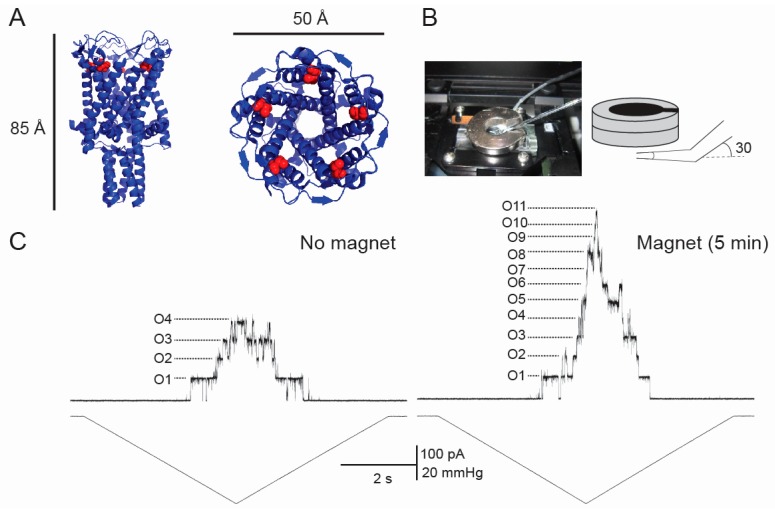
The effect of static magnetic field (SMF) on the gating of the M42C mechanosensitive channels of large conductance (MscL) labelled with paramagnetic nanoparticles coated with sulfhydryl (SH) groups. (**A**) 3D crystallographic structure of MscL viewed from the side (left) and the top (right). Red spheres show the methionine 42 residue of each subunit, which is replaced with a cysteine used for labelling in this study; (**B**) patch-clamp chamber with a ring-shaped magnet (left) and SMF application protocol (right); (**C**) patch-clamp recording from M42C MscL (upper trace) upon application of a pressure ramp (lower trace) in the absence of the magnetic field (left) and 5 min after SMF application (right; same patch). The number of the open channels (O1–11) is indicated in the trace.

In our previous studies we used the bacterial MS channel of large conductance (MscL) as a model channel and demonstrated that static magnetic fields of 400 mT affected the activity of MscL reconstituted into liposome bilayers [23]. In subsequent ACEBR research, we developed superparamagnetic CoFe_2_O_4_ nanoparticles and labelled them by sulfhydryl (SH) groups for attachment to M42C mutant MscL. Activation of MscL by a 300 mT magnetic field with the nanoparticles attached to the channel was examined in patch clamp experiments, with the SMF increasing the number of channels activated by pressure applied to patch pipettes (Figure 1) [24]. These results need to be further investigated given that, in addition to the effects they exert on diamagnetic membrane bilayers, SMFs could also affect MS channels in human cells via the strongly ferromagnetic magnetite/maghemite found in the human brain [25].

To complement these in vitro experiments [26], ACEBR has also been employing computer simulations [27] to investigate the influence of SMFs on the structural integrity of lipid membranes, mimicking phospholipid composition of neuronal membranes in the brain tissue. This provides a powerful means of assessing theoretical interactions that may impact on more complex in vitro and in vivo scenarios. Specifically, the effects of 0.2–1 T SMFs on the surrounding water, individual lipid molecules and the overall lipid-bilayer anisotropy will be studied. This visualisation and quantitative characterisation of the structural changes at the modelled molecular level will allow us to identify the field strengths causing the membrane response and improve our fundamental understanding of the magnetic field influences on biomolecules, allowing us to determine the likelihood of adverse impacts on biological functioning.

### 4.3. Super High Frequency EMF Effects on Membrane Electroporation

At extremely high field strengths, we have shown that 18 GHz continuous wave exposure has sterilisation/inactivation effects on cells that are not easily explained by bulk temperature increase alone [28]. This research stream is exploring the EMF–bioeffect relation in detail to better understand the interaction mechanism(s) and potential application of the EMF.

Accordingly, ACEBR has been studying the effects of super high frequency (18 GHz) EMF exposure on typical representatives of prokaryotic and eukaryotic taxa, including three Gram-negative bacteria (*Bacillus subtilis* NCIMB 3610T, *Branhamella catarrhalis* ATCC 23246, and *Escherichia coli* ATCC 15034), six Gram-positive bacteria (*Kocuria rosea* CIP 71.15T, *Planococcus maritimus* KMM 3738, *Staphylococcus aureus* CIP 65.8T, *Staphylococcus aureus* ATCC 25923, *Staphylococcus epidermidis* ATCC 14990T, and *Streptomyces griseus* ATCC 23915), a eukaryotic unicellular organism (yeast *Saccharomyces cerevisiae* ATCC 287), and red blood cells obtained from a New Zealand rabbit. Three EMF exposure parameters were varied (power, duration, and exposure repetitions) to determine their effect on cell permeability related processes. Advanced microscopy techniques were employed, comprising scanning electron microscopy (SEM), transmission electron microscopy (TEM), and confocal scanning laser microscopy (CSLM) together with fluorescent probes, in order to allow a thorough examination of cell membrane morphology and permeability following EMF exposure(s).

This research determined, for the first time, that regardless of the differences in cell wall/membrane structures, exposure to 18 GHz EMF induced cell permeabilisation, as confirmed via the ability of the cells to uptake silica nanospheres (23 nm and 46 nm in diameter), in all of the cell types studied, in a manner that could not be duplicated using conventional heating methods under similar bulk temperature conditions [29,30,31]. Moreover, a large proportion of the cells remained viable (85%) throughout the exposures (excluding erythrocytes) as confirmed directly using the colony-forming units counting technique. Cells remained permeable for at least nine minutes after EMF exposures. A dosimetry analysis revealed that the EMF exposure required to induce cell permeation such that the membrane was able to uptake 46 nm nanospheres was between three and six one-minute EMF exposures with a specific absorption rate (SAR) of 5 kW/kg and 3 kW/kg per exposure, respectively, depending on the cell types being studied. These results are important in that the membrane effects do not occur with Peltier plate-induced equivalent bulk temperature increases, and cannot be explained via traditional electroporation mechanisms, which require brief pulsed fields [32]. It is hypothesised that the taxonomic affiliation and cell wall/membrane structures (e.g., the presence of peptidoglycan layer, mannoprotein/β-glucan layer, phosphatidyl-glycerol and/or pentadecanoic fatty acid) may affect the extent of permeabilisation to allow the uptake of 46 nm nanospheres [29,30]. However, precisely how this relates to EMF itself is not clear.

To clarify this hypothesis, ACEBR has been employing computational molecular dynamics simulations to study the effects of 18 GHz EMF on the structure and dynamics (fluidity) of lipid membranes. The simulations provide an atomistic insight into the lipid bilayer response to electric fields of different intensities, where the fluidity of the lipid bilayer and structuring of surrounding water are characterised in a level of detail that is not experimentally achievable. The initial lipid bilayer model in this work consists of 400 POPC (1-palmitoyl-2-oleoyl-sn-glycero-3-phosphocholine) lipids (200 in each leaflet) with 150 mM NaCl and explicit water molecules, with the modelled effect of EMF enabling us to identify specific combinations of field intensities and frequencies required to achieve field-induced biomembrane permeability. It is anticipated that the results will provide the mechanistic understanding required to better guide the in vitro and future in vivo work, as well as informing potential biomedical applications in biomedical engineering, gene therapy, and drug delivery.

### 4.4. Cellular and Modelling Work on Gene and Protein Expression

As described above, a number of studies have reported important biological effects of MC-EMF at levels within ICNIRP guideline restrictions (SAR 2.0 W/kg), which, if accurate, would contradict the prevailing scientific view. These include effects of 800–2400 MHz EMF and extremely low-frequency pulsed EMF on global gene and protein expression in a range of cell types [33,34,35]. ACEBR is conducting in vitro studies to replicate and potentially delineate the conditions required for such effects to occur. In support of this, in silico studies are also being performed to model the effects of static fields on protein conformation using molecular dynamics simulations. Our current experimental studies suggest that low level MC-EMF may produce opposing effects depending on EMF frequency and power. Future research will include replication, as well as irradiation of TRP and Piezo ion channel proteins expressed in different cell cultures. This line of research will develop a better understanding of the relation between MC-EMF exposure and health, but also a set of methodologies that could be utilised to evaluate the safety of future MC-EMF technologies.

## 5. Neurodegenerative Diseases

Alzheimer’s disease, the most common form of dementia, is a relentlessly progressive neurological disorder characterised clinically by cognitive deterioration and behavioural disturbances, and pathologically by neuronal neurofibrillary tangles comprised of tau protein and extracellular amyloid (Aβ) deposits within senile (neuritic) plaques. The pathogenesis is poorly understood, and effective preventive and treatment regimens have remained elusive. Since mobile phones are held close to the head, the brain typically receives higher exposures than other body tissues. Although this has led to considerable research looking for negative effects on the brain, recent studies [36,37] suggest that such exposure could be beneficial in reducing brain amyloid deposition and improving cognition in Alzheimer’s disease. In addition to the potential health benefit that this line of research might produce, it is also important as the findings contrast with the prevailing scientific consensus, which holds that low-level MC-EMF does not affect health, either adversely or beneficially.

### 5.1. Animal Studies

Two independent studies have now reported that long-term (7–8 months) MC-EMF exposure reduces brain amyloid deposition and improves cognition in transgenic mouse strains of Alzheimer’s disease [36,37]. Similar results have also been found after scanning ultrasound exposure of the brains of the same strain of transgenic mice [38]. In order to replicate and extend these findings, we have neuropathologically characterised an APPswe/PS1dE9 transgenic mouse model of Alzheimer’s disease, where abundant amyloid deposition was found in the mice, and now aim to determine whether protracted 2.45 GHz 3 G exposure in a reverberation chamber (2 h per day, for 8 months, starting at 1.5 months of age) can reduce cerebral amyloid deposition in this “amyloid-only” mouse strain, and impede the development of cognitive deficits. It will also include a thermal (non-EMF) control condition to determine whether thermal changes are responsible for the reported effect (as might be hypothesised based on the similarity of findings in the EMF and ultrasound studies). If this study supports the findings of previous research, it could potentially lead to the development of novel EMF-based preventive intervention strategies for Alzheimer’s disease.

### 5.2. Theoretical Molecular Modelling

It is believed that high-level fields of specific frequency can excite certain vibrational modes of proteins and other biomolecules, causing structural changes which can alter conformation and, ultimately, function [39,40,41]. These conformation changes could, in principle, lead to misfolding and affect aggregation of proteins into insoluble amyloid fibrils, a process responsible for many debilitating and age-related diseases, such as Alzheimer’s disease described above. If a better theoretical understanding of such effects and their relations with disease processes could be obtained, this would help clarify whether/how MC-EMF exposure could, in principle, affect certain neurodegenerative processes.

Our previous molecular modelling studies of the effects of electric fields on insulin [42,43,44,45] demonstrated that by modulating field parameters (strength/frequency) of the signal, it is possible to achieve protein alignment along the field direction, or completely alter the protein’s native conformation. This work suggests that EMFs can, in principle, influence protein alignment (e.g., in the amyloid fibrils). Based on this and the recent experiments suggesting that MC-EMF can affect amyloid fibrils [46], we undertook a new theoretical modelling study where we systematically varied the field parameters in order to identify and characterise any molecular-level effects on amyloid protein structure [47], using all atom molecular dynamics simulations. Our model protein was ApoC-II, an amyloidogenic lipoprotein implicated in heart disease. We previously studied this under various conditions [48,49,50], and so it can be used as a benchmark to determine any effects that MC-EMFs may have on peptide structure and dynamics. In this study we applied field strengths varying from 0.7 to 0.0007 V/nm to identify the lowest field strength at which we can reproducibly detect and characterise the field effects on the structure and dynamics of the protein models.

**Figure 2:**
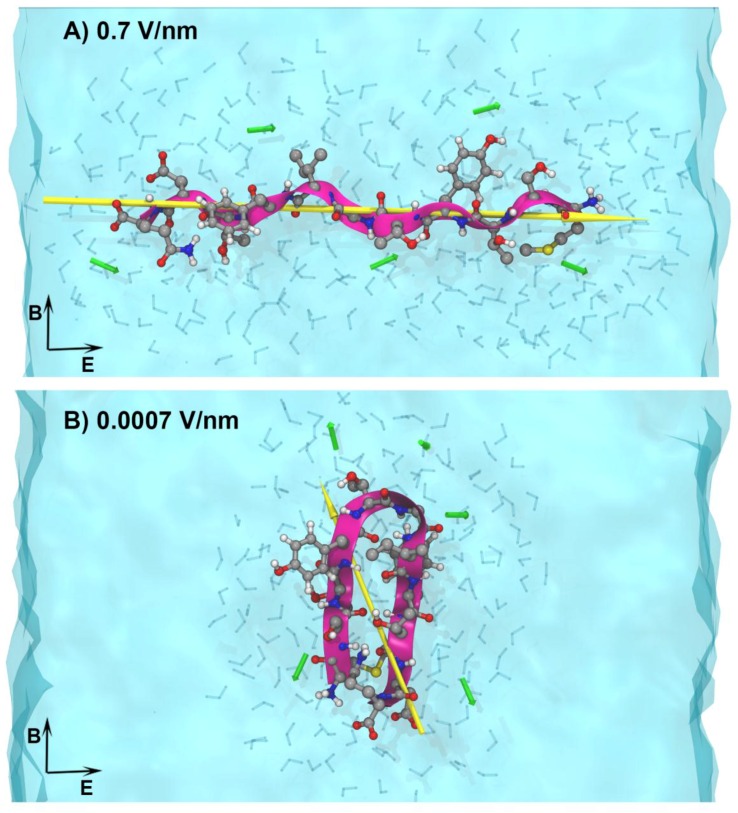
All-atom computer model of an amyloid peptide in solution under the influence of varying strength electromagnetic fields (EMF) (from [47]), (**A**) exemplifies the amyloidogenic ApoC-II peptide adopting an elongated conformation under the fields higher than 0.04 V/nm due to the peptide dipole alignment along the electric field direction; (**B**) illustrates the peptide conformation being native-like under the fields less than 0.04 V/nm in strength. For detailed systematic analysis of the EMF field strength effects on the conformational dynamics of this peptide refer to [47]. The peptide conformation is shown as a ribbon (purple) and atomic structure in CPK representation. Water molecules near the peptide are shown explicitly in grey CPK representation and the overall solution is coloured blue. Yellow arrows represent the peptide dipole moment and black arrows represent the direction of the applied electric and magnetic field respectively.

We found that high-strength EMFs altered the structure and dynamics of the amyloidogenic peptide by elongating and aligning it along the electric field direction (Figure 2A. The findings suggest that there may be a “window” of EMF strengths at which the amyloid structure can be destabilised and potentially destroyed. It is important to note that we found no measurable effects, within the timeframe of our simulation, below the exposure level of 0.0007 V/nm (Figure 2B). These exposures are many orders of magnitude higher than those from commercial telecommunication devices. We are currently investigating the effects of MC-EMF frequencies in the 1.0–2.45 GHz range on the structure and dynamics of the ApoC-II-derived peptide at the lowest field intensity level at which the effects were theoretically measurable [47].

Furthermore, in light of the recent reports by Arendash et al. [36,46], and to complement the in vitro work described above, we are currently using classical all-atom molecular dynamics simulations to better understand the role of EMF on the structure of the amyloid-β 40 (Aβ40) and 42 (Aβ42) proteins implicated in Alzheimer’s disease. Recent studies suggest that the trimer and tetramer forms of the Aβ protein are the toxic species responsible for Alzheimer’s disease and neuron death [51,52]. Using the available all-atom 3D structure of the Aβ fibril [53], we are currently performing molecular simulations to investigate the effects of field strength and frequency on the structure and stability of the monomeric and oligomeric forms of the Aβ protein.

## 6. Dosimetry

Dosimetry research is aimed at improving estimates of electromagnetic energy deposited into specific locations within biological systems, including (but not limited to) humans, and is of critical importance in the design and interpretation of bioelectromagnetic experiments [2]. ACEBR is engaged in a number of ongoing projects relating to dosimetry research gaps that were designated high priority research items in the WHO 2010 RF Research Agenda.

### 6.1. Occupational Thermoregulation Modelling

Human workers, such as defence personnel and telecommunications engineers, are sometimes exposed to high levels of MC-EMF while working in harsh environments and wearing protective clothing that reduces heat loss. However, EMF standards have relied heavily on thermal modelling work that has used nonrealistic conditions, such as unclothed personnel in temperate climates, and therefore may underestimate associated increases in body core temperature. ACEBR is attempting to correct this by modelling the effect of MC-EMF on thermoregulation in realistic exposure scenarios. Preliminary results indicate that the standards offer adequate protection for the harshest of climates, but that the safety margins may not be as large as previously thought [54]. Work is continuing to determine, across the frequency spectrum, whether the safety margins may be further compromised by certain characteristics of stature associated with variations in body morphology, or by more intense local exposures (which are allowable under the present ICNIRP guidelines).

### 6.2. Terahertz Frequencies and Modelling of Absorption

Terahertz (THz) frequencies in the range of 0.1–100 THz are now being increasingly exploited for telecommunications and biomedical applications. However, the electrical characteristics of biological tissue in this range, with particular emphasis on energy absorption in bacterial spores and human skin, are largely unknown. Recent ACEBR work on bacterial spores [29] indicates a possible method of infection control using THz exposure, and this work is aimed at further modelling THz absorption patterns in tissue. Given their increasing use, this work will help to determine whether the THz EMF frequencies could adversely impact on biological function and human health.

### 6.3. Improving Modelling of Temperature in Tissues

The effect of raised temperature on electrical parameters used in advanced modelling of tissue energy absorption is important for the improvement of precision of catheter-delivered hyperthermia and ablation procedures. The accuracy of modelling of temperature rise in tissues is improved by compensating for temperature-induced changes in dielectric constants and conductivity. This study is improving the prediction of the consequences of these changes, with the aim to improve the efficiency of hyperthermic therapies, including RF ablation.

### 6.4. Characterisation of RF Exposure in Public

The electromagnetic environment in everyday life continues to become more varied and perhaps more uncertain, making accurate predictions of MC-EMF exposure in public spaces due to Wi-Fi, phone base-station, and neighbouring handset transmitters increasingly difficult. ACEBR is conducting an ongoing survey which is aimed at producing better MC-EMF exposure characterisation of everyday activities, such as attendance at sporting fixtures, shopping malls and travel on public transport. The rollout of public Wi-Fi and increase of exposures due to communication technologies in general make this type of survey invaluable in informing the public on actual exposure levels, and ensuring compliance with international guidelines.

## 7. Risk Communication

Regardless of whether MC-EMF affects health, communicating resultant risk assessments is seen as an important part of any risk management process, in part because many problems and misunderstandings can arise due to miscommunication. The overarching goal of this ACEBR program is to contribute to more informed judgments by better risk communication, with its focus on understanding the effect of *precautionary messages*. Precautionary messages are often used by health agencies to provide information about behavioural measures that can be used in order to reduce risk, in situations where risk is uncertain. These are not necessarily designed to increase concern, but research has shown that this is indeed an unintended effect of precautionary messages regarding MC-EMF [55]. In a study designed to better understand the role of recipient characteristics in the precautionary message effect, 298 university students completed a questionnaire with either factual information concerning potential health effects of MC-EMF, or precautionary statements in addition to this information. In addition to replicating previous research in that precautionary messages increased how threatened respondents felt about their MC-EMF environment, results demonstrated that this effect was primarily due to those with low levels of trait anxiety (precautionary messages in low-anxious people increased threat perception relative to that of the high-anxious people), with this effect larger in women than men [56]. This suggests that individual differences are important determinants of the success of risk communication strategies, further complicating the difficulties of communicating MC-EMF risk information. ACEBR is also exploring the characteristics of the message that leads to this effect, as detailed in this special edition [57].

## 8. Conclusions

Given the ubiquitous nature of MC-EMF in our modern environment, it is essential that we continue to conduct the highest quality research to investigate any possible effects that such exposures could have on human health and wellbeing. The current stream of research being undertaken by ACEBR is addressing the most important aspects of the MC-EMF health debate, with a strong focus on mechanisms, neurodegenerative diseases, cancer, and exposure dosimetry. This research takes the current scientific status quo as its premise, but also reaches beyond that in questioning the premises upon which it is based. It is also complemented by the ACEBR risk communication stream, which aims to ensure that whatever is found is communicated effectively and appropriately.
